# Lifespan oscillatory dynamics in lexical production: A population-based MEG resting-state analysis

**DOI:** 10.1162/imag_a_00551

**Published:** 2025-04-28

**Authors:** Clément Guichet, Sylvain Harquel, Sophie Achard, Martial Mermillod, Monica Baciu

**Affiliations:** Université Grenoble Alpes, CNRS LPNC UMR 5105, Grenoble, France; Université Grenoble Alpes, CNRS, INRIA, Grenoble INP, LJK, Grenoble, France; Neurology Department, CMRR, Grenoble Hospital, Grenoble, France

**Keywords:** language, magnetoencephalography, cognitive aging, Default Mode Network, Hidden Markov Model, DECHA

## Abstract

Lexical production performances have been associated with cognitive control demands increase with age to support efficient semantic access, thus suggesting an interplay between a domain-general and a language-specific component. Current neurocognitive models suggest the Default Mode Network (DMN) and Fronto-Parietal Network (FPN) connectivity may drive this interplay, impacting the trajectory of production performance with a pivotal shift around midlife. However, the corresponding time-varying architecture still needs clarification. Here, we leveraged MEG resting-state data from healthy adults aged 18–88 years from a CamCAN population-based sample. We found that DMN-FPN dynamics shift from anterior-ventral to posterior-dorsal states until midlife to mitigate word-finding challenges, concurrent with heightened alpha-band oscillations. Specifically, sensorimotor integration along this posterior path could facilitate cross-talk with lower-level circuitry as dynamic information flow with more anterior, higher-order cognitive states gets compromised. This suggests a bottom-up, exploitation-based form of cognitive control in the aging brain, highlighting the interplay between abstraction, control, and perceptive-motor systems in preserving lexical production.

## 1 Introduction

The increase in life expectancy is associated with a stringent need to understand the contributing factors of healthy and “successful” aging (United Nations, 2023). Research in cognitive aging has uncovered significant insights into the natural progression of cognitive functions with age. While several cognitive abilities, including episodic and working memory, attentional and inhibitory control, begin to decline in early adulthood (Salthouse, 2019), language skills remain relatively preserved (Baciu et al., 2016;Shafto & Tyler, 2014). Given the fundamental role of language in human cognition (Hagoort, 2023;Pinker, 2008), gaining a deeper understanding of the neural mechanisms that support the maintenance of language functions is essential.

Relatedly, studies suggest that language interacts across various cognitive domains, potentially as the common thread behind neurocognitive changes supported by an intricate interplay of perceptive-motor and cognitive systems (Fedorenko et al., 2024;Harada et al., 2013;Hartshorne & Germine, 2015;Saryazdi et al., 2019). For example, the Language-union-Memory (L∪M) framework (Roger, Banjac, et al., 2022) proposes that language processing involves the bidirectional flow of information between external sensory modalities and long-term memory, flexibly monitored by task-relevant control processes. A previous task-fMRI meta-analysis has shown that language processes, including lexical production, are distributed across the cortex across subsystems that extend beyond core language regions (Malik-Moraleda et al., 2022), overlapping with the boundaries of control-executive, abstract-conceptual (Kurmakaeva et al., 2021), and sensorimotor networks at rest (Roger, Rodrigues De Almeida, et al., 2022). Relatedly, data-driven classification of cortical and subcortical resting-state fMRI activity highlighted that even core language regions bind together in a coherent language network (Ji et al., 2019), suggesting that neural processes relevant to core language processing can also be investigated at rest.

Overall, this study assumes that the resting-state architecture provides a general framework that complements task-based investigations of lexical production, in line with the idea that language production is an inter-cognitive function supported by multiple cortical networks. Changes in hemodynamic activity within these networks have allowed for a comprehensive description of the mechanisms involved in preserving language function across the lifespan. For example, the Lexical Access and Retrieval in Aging or LARA model (Baciu et al., 2021;Baciu & Roger, 2024) posits domain-general and language-specific compensatory mechanisms, reflecting the interplay between executive functioning and semantic memory.

In this context, longer naming latencies and more frequent tip-of-the-tongue situations with age could be mitigated by enhanced semantic access, thanks to a distributed anatomo-functional reorganization (Alves et al., 2023;Guichet, Banjac, et al., 2024;Guichet, Roger, et al., 2024;Kavé & Mashal, 2012). Specifically, the enrichment of semantic repositories (Cutler et al., 2025;Gollan & Goldrick, 2019;Salthouse, 2019) could represent a strategy for maintaining the retrieval of words with stronger semantic connections (Benítez-Burraco & Ivanova, 2023) and conceptual categorization (Alves et al., 2021;Kiran, 2007) contingent upon accumulated vocabulary knowledge throughout adulthood (Kavé & Halamish, 2015;Shafto et al., 2017). However, more pronounced inhibition deficits could challenge the ability to ignore task-irrelevant information (Blanco et al., 2016;Hoffman, 2018;Spreng & Turner, 2019), leading to longer searches through a growing semantic store (Cutler et al., 2025). Indeed, reduced access and retrieval of lexico-semantic representations (Baciu et al., 2016;Boudiaf et al., 2018) may induce more automatic and distracting thoughts, leading to a global decline in selecting the appropriate semantic information (Badre & Wagner, 2007;Hoffman & MacPherson, 2022;Jefferies, 2013;Martin et al., 2022), with difficulties beginning around midlife (Benítez-Burraco & Ivanova, 2023;Shafto & Tyler, 2014;Verhaegen & Poncelet, 2013).

At the brain level, the central role of semantic control in lexical production is reflected by activations within the multiple demand (MD) and default mode network (DMN). The MD network (Duncan et al., 2020) is composed of a core set of regions from the fronto-parietal (FPN), dorso-attentional (DAN), and cingulo-opercular (CON) networks that are relevant for goal-directed cognition (Camilleri et al., 2018;Cieslik et al., 2015;Langner & Eickhoff, 2013;Rottschy et al., 2012). The DMN, usually activated in the absence of external stimuli, plays a crucial role in self-directed and semantically rich cognitive processes (Alves et al., 2019;Menon, 2023;Raichle et al., 2001). Importantly, semantic control regions appear to adjust their activation to cognitively demanding semantic processes by engaging a set of areas “sandwiched” between the MD and DMN (Chiou et al., 2023). This aligns with current models (Ralph et al., 2017;Reilly et al., 2023) delineating a control subsystem involving the anterior-ventral parts of the IFG (Branzi & Lambon Ralph, 2023) and pMTG (Jefferies, 2013;Noonan et al., 2013) and a language-specific subsystem primarily storing multimodal representations in the ventral ATL (Hoffman & Morcom, 2018;Jackson, 2021).

In the context of healthy aging, models such as the Default-Executive-Coupling Hypothesis of Aging (DECHA) propose that lateral PFC engagement within the FPN is functionally coupled with DMN suppression (Spreng & Turner, 2019), thus integrating domain-general and language-specific mechanisms central to semantic control. Evidence supports that DMN suppression optimizes goal-driven executive tasks, including semantic classification (Lustig et al., 2003), by reducing self-directed processes such as mind wandering (Anticevic et al., 2012;Buckner & DiNicola, 2019;Kamp et al., 2018;Leonards et al., 2023). In older ages, reduced DMN suppression may lead to a stronger and more inflexible DMN-FPN coupling that impacts externally directed task performances. However, when prior knowledge is congruent with the task, increased anterior DMN connectivity (right ATL-mPFC) in older adults has been found to strengthen verbal semantics and on the contrary impair the ability to discern complex semantic relationships that require more top-down control (Krieger-Redwood et al., 2019), such as lexical production (Guichet, Banjac, et al., 2024). Thus, it seems that a flexible DMN-FPN coupling that can adapt to internal and external tasks is key to maintain goal-directed behavior with age. Nonetheless, to our knowledge, the age-related mechanisms underlying this flexibility have only been examined through the prism of time-averaged fMRI studies, although control components such as inhibition are temporally focused (Courtney & Hinault, 2021).

Moreover, recent teamwork suggested that, as the DMN becomes less deactivated and DMN-FPN flexibility decreases, older adults may switch toward a more “energy-efficient” strategy of integration based on short-distance functional connections within lower-level networks (i.e., SMN, CON). This strategy could exploit semantic memory-guided control inputs for lexical production (Guichet, Banjac, et al., 2024). However, the hypothesized orchestration between lower-level networks in older adults also remains unclear.

In this study, we suggest that a time-varying approach is more adequate to investigate such age-related effects. In that regard, magnetoencephalography (MEG) offers a higher temporal resolution with comparable resting-state functional connectivity, as measured with fMRI (Sadaghiani & Wirsich, 2020), making this modality a strong candidate for examining moment-to-moment inter-regional synchrony, or dynamic FC.

Propagating brain rhythms are an essential aspect of dynamic FC that cannot be derived by conventional functional MR imaging as resting-state fMRI usually captures low-frequency spontaneous fluctuations of BOLD activity. Although the exact anatomical and functional factors that contribute to the emergence of brain oscillations are still the object of intensive research (Buzsáki & Vöröslakos, 2023), prior work has demonstrated that key cortical networks have distinct neurophysiological signatures (Keitel & Gross, 2016). Previous studies have associated lower-level sensory processing to beta and gamma-range activity (e.g., beta band for sensorimotor regions;Davis et al., 2012;Heinrichs-Graham et al., 2018), while prominent delta, theta, and alpha spectral peaks have recently been shown to reflect higher-level processes in transmodal cortices (i.e., DMN, FPN) (Royer et al., 2025). Delta oscillations are associated with, but not exclusively to, inhibitory control over external stimuli in a state of inner focus; similarly theta oscillations are notably linked with the hippocampus, supporting its involvement in memory (Rempe et al., 2023).

With age, several studies linked declines in perceptual speed and executive functioning to reduced delta and theta power at rest (Gómez et al., 2013;Leirer et al., 2011;Ustinin et al., 2025;Vlahou et al., 2014). Importantly, alpha activity is often presented as the backbone of functional dynamics and inhibitory process (Jensen & Mazaheri, 2010;Klimesch et al., 2007), allocating attentional resources and regulating brain activation (Courtney & Hinault, 2021). In the context of our study, enhanced alpha band synchrony in FPN regions with age could reflect efforts to maintain interference control as the DMN becomes more engaged.

With age, several studies linked declines in perceptual speed and executive functioning to reduced delta and theta power at rest (Gómez et al., 2013;Leirer et al., 2011;Ustinin et al., 2025;Vlahou et al., 2014). Importantly, alpha activity is often presented as the backbone of functional dynamics and inhibitory process, allocating attentional resources and regulating brain activation (Courtney & Hinault, 2021). In the context of our study, enhanced alpha band synchrony in FPN regions with age could reflect efforts to maintain interference control as the DMN becomes more engaged.

Overall, the main goal of this study was to provide an integrated spatial, temporal, and spectral description of the mechanisms involved in maintaining language function across the lifespan, specifically considering the time-varying dynamics of canonical resting-state networks such as the DMN and FPN. We hypothesized that DMN oscillatory activity will drive the dynamic resting-state architecture, maintaining lexical production performance according to two mechanisms: (i) a Domain-General (DG) mechanism engaging bilateral prefrontal cortices correlated with flexible abilities (i.e., problem-solving). We also expected more infrequent activations in the DMN-FPN states that support younger-like lexical production performances, reflecting a shift toward more inflexible DMN-FPN states in older adults as predicted by the DECHA model (Spreng & Turner, 2019). This inflexibility could be characterized by heavier alpha-band synchrony, reflecting greater top-down control demands for semantic word retrieval (Zioga et al., 2024). (ii) a Language-Specific (LS) mechanism supporting access to lexico-semantic representations within lateral and mesial left-lateralized frontotemporal networks (Baciu & Roger, 2024). We expected this mechanism to peak in midlife, reflecting heightened information flow in brain states implementing DMN-CON coupling (Guichet, Banjac, et al., 2024).

## 2 Material and Methods

**Fig. 1.**
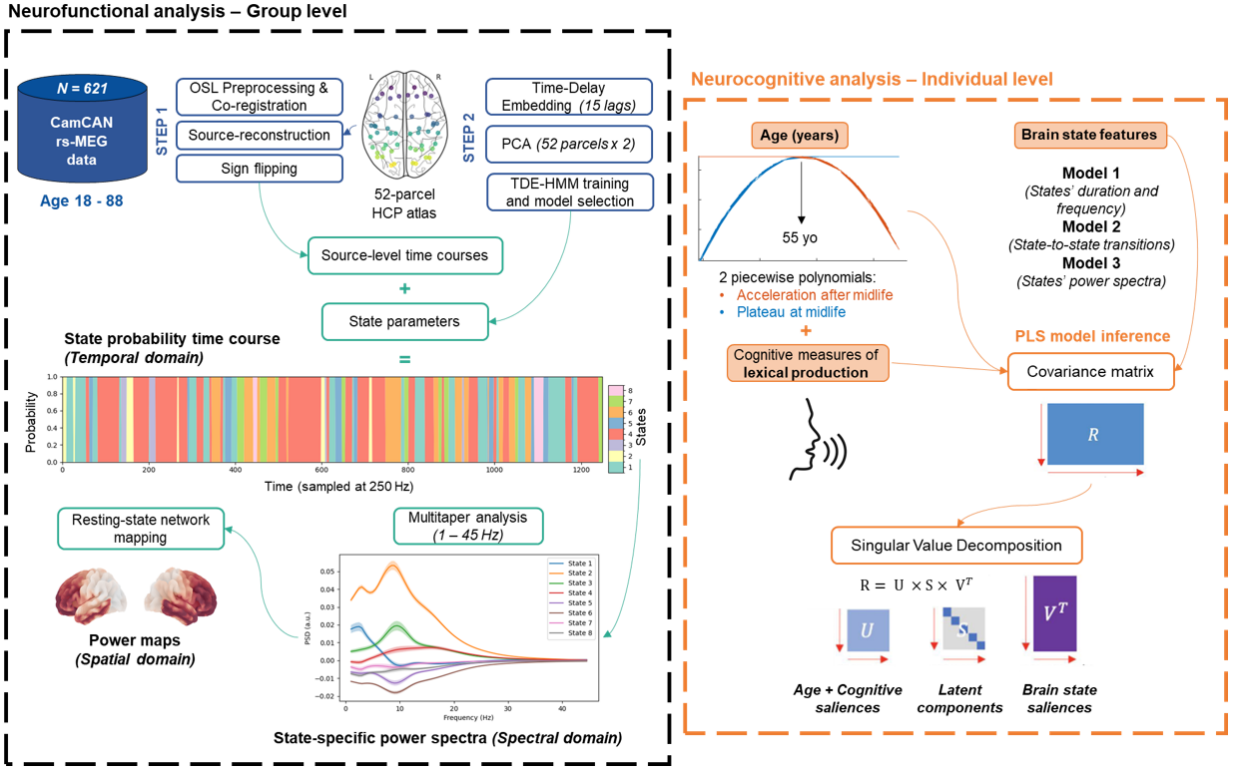
Overview of the analysis pipeline. (*Neurofunctional*). Following the preprocessing steps, we employed the Time-Delay Embedded Hidden Markov Model (TDE-HMM) to identify functional brain states from resting-state MEG time series data. We then extracted each state’s group-level temporal, spectral, and spatial features (*Neurocognitive*). We examined the relationship between age-related lexical production performance and brain state features using the Partial Least Squares (PLS) technique. Age was parametrized using two piecewise polynomials centered at 55 years old to explore non-linear trajectories during inference. Cognitive performance was assessed with eight tasks directly or indirectly related to lexical production. We further considered 3 types of brain features in 3 distinct models: temporal metrics (duration & frequency), transition probabilities from one state to another, and the power spectra of the top 20 state-relevant parcels. For details, refer toSection 2.

We leveraged MEG resting-state data from healthy adults aged 18–88 years from the CamCAN population-based cohort (Cam-CAN et al., 2014) and assessed lexical production performance using eight direct or indirect cognitive measures. The study analysis pipeline is completely unsupervised and data driven: (i) First, we identified dynamic brain states common to all individuals using the Hidden Markov Model (HMM); (ii) second, we related these states’ spatial, temporal, and spectral features to lexical production performance across the lifespan.Figure 1presents an overview of the pipeline.

### 2.1 Participants and data

#### 2.1.1 Participants

Participants for MEG analysis were 621 healthy adults (306 females, 315 males) aged 18.5–88.9 years at the time of neuropsychological assessment. All participants gave written informed consent as part of the Cambridge Center for Ageing and Neuroscience project following approval by the Cambridgeshire 2 Research Ethics Committee (reference: 10/H0308/50) (Cam-CAN et al., 2014).

To evaluate the brain–behavior relationship, we excluded 24 participants with more than 3 missing cognitive scores among the 8 considered in this study. Additionally, 130 participants without education-related information were excluded from the study (refer toSection 2.1.3). The final sample size comprised 467 participants (237 females and 230 males). Eventual missing scores were imputed by the median value matched to the age decile of the participant.

#### 2.1.2 Magnetoencephalographic (MEG) data

MEG data were collected in a magnetically shielded room in a 306-channel Vectorview MEG system (Elekta Neuromag), 102 magnetometers, and 204 orthogonal planar gradiometers (Taylor et al., 2017). A resting-state scan (participants with eyes closed) data ran for 8 min 40 s and sampled at 1 kHz (high-pass filter of 0.03 Hz). The MaxFilter 2.2.12 software (Elekta Neuromag, Helsinki, Finland) was used to apply temporal signal space separation (tSSS) (Taulu & Simola, 2006) for noise reduction and offline head motion correction based on the Head-Position Indicator (HPI) coils used to estimate the head position within the MEG helmet.

#### 2.1.3 Cognitive data

Using eight neuropsychological tests, lexical production performance was measured considering domain-general (DG) and language-specific processes (seeTable 1). Education levels were also considered as previous work suggested that this factor modulates semantic retrieval (Shafto et al., 2017). Education attainment was mapped to a 10-point scale ranging from CSE/GCSE diploma to PhD. To keep homogeneity among participants, we considered the highest degree obtained at age 30 years.

**Table 1.** Neuropsychological tasks and associated cognitive processes were considered in this study.

Neuropsychological test	Cognitive processes	Task type and relation to lexical production
Picture naming	Language (phonological and semantic access)	LP, Direct
Verbal fluency	Language (phonological and semantic access) and EF	LP, Direct
Tip-of-the-tongue situations	Language (retrieval) and EF (monitoring)	LP, Direct
Proverb comprehension	Language (comprehension) and EF (abstraction)	Language, Indirect
Sentence comprehension	Language (syntax, semantic)	Language, Indirect
Story recall	Long-term memory	Language, Indirect
Cattell task	EF (Logical reasoning, problem solving)	Domain-general, Indirect
Hotel task	EF (multitasking, planification, self-monitoring)	Domain-general, Indirect

A detailed description can be found inSupplementary Materials. LP = Language production.

### 2.2 MEG data processing

#### 2.2.1 Preprocessing and co-registration

MEG analysis steps were performed with the OHBA Software Library (OSL) in Python 3.8.16 following OSL guidelines (https://github.com/OHBA-analysis/osl). Preprocessing began by discarding the first 30 s of the raw signals before bandpass filtering using a 5^th^-order IIR Butterworth filter [0.5–125 Hz]. Notch filtering was also applied at 50 Hz, 88 Hz, and harmonics (notch width = 2 Hz). This step suppressed interference with power line noise and known artifacts specific to the CamCAN dataset. Subsequently, the data were downsampled to 250 Hz, and automated detection of bad segments and channels was conducted using the generalized-extreme studentized deviate (G-ESD) algorithm (Rosner, 1983). Further denoising was performed with a FastICA decomposition (Hyvarinen, 1999), decomposing signals into 64 components and rejecting ECG/EOG-related artifacts. Finally, wrong channels were interpolated from ICA-cleaned data using spherical spline interpolation (Perrin et al., 1989).

The resulting MEG data were co-registered to each participant’s structural T1-weighted MR image with an iterative close-point algorithm (ICP) that matched digitized anatomical fiducial points (nasion and bilateral pre-auricular points) to the scalp. The scalp’s surfaces, inner skull, and brain were extracted with FSL’s Brain Extraction Tool (BET).

#### 2.2.2 Source reconstruction and sign flipping

Following OSL guidelines, preprocessed sensor data were bandpass filtered [1–45 Hz], and sources were reconstructed onto an 8 mm isotropic dipole grid. This reconstruction was based on a single-shell lead-field model in MNI space and employed a linearly constrained minimum variance (LCMV) scalar beamformer (Van Veen et al., 1997;Woolrich et al., 2011). Continuous time series were extracted and parcellated into 52 regions derived from the HCP-MMP 1.0 atlas (Glasser et al., 2016), recently adapted for MEG analysis (Kohl et al., 2023). To mitigate source leakage, we applied the symmetric multivariate leakage reduction algorithm (Colclough et al., 2015). To address inconsistencies across channels and subjects in source-reconstructed dipole signs, we employed a validated sign-flipping algorithm (Vidaurre et al., 2018).

### 2.3 Hidden Markov Model

This study employed the Time-Delay Embedded variant of the Hidden Markov Model (TDE-HMM) to provide a spatially, spectrally (i.e., defined as a function of frequency), and temporally resolved (i.e., state is active or inactive) description of MEG resting-state neural activity (Vidaurre et al., 2018). Data preparation, model training, and post hoc analysis were carried out with the toolbox*osl-dynamics*(Gohil et al., 2023) in Python 3.10.14.

#### 2.3.1 HMM description

The HMM posits that the observed MEG data can be generated from a sequence of hidden brain states whose activations wax and wane over time. The TDE variant specifically models the autocovariance of the signal around time point*t*, that is the covariance between*t*and a time-lagged version of itself*t+L*where*L*defines the time window around*t*. Thus, this embedding may better account for the conduction delay between communicating brain regions (Hussain et al., 2023). Mathematically, the embedded space is described using a zero-mean Gaussian distribution (Masaracchia et al., 2023):

$$P(Y_{t})\sim N\left(0,\Sigma \right),\forall t.$$

Here,$Y_{t}=(y_{t-L},...,y_{t},...,y_{t+L})$represents a linear combination of time points*t*for a multichannel time series*y*, and*Σ*is the multivariate autocovariance matrix.

The TDE-HMM is then defined as

$$P(Y_{t}|x_{t}=k)\sim N\left(0,\Sigma^{\left(k\right)}\right),$$

where$x_{t}$is a hidden variable indicating the state*k*active at time point*t*and$\Sigma^{\left(k\right)}$is the autocovariance matrix describing the spectral content of the state.

#### 2.3.2 Time-Delay Embedding (TDE)

In line with previous work (Huang et al., 2024;Vidaurre et al., 2018), we prepared source-space MEG data by embedding*L*= 15 lags evenly distributed across the range -125 to 125 Hz around each time point*t*. Thus, considering 52 parcels, the embedding resulted in 780 channels with 303,810 (780*779/2) parameters to estimate for each state autocovariance matrix. To mitigate overfitting concerns and decrease computational cost, principal component analysis (PCA) was applied to reduce the dimensionality of the embedded space to 104 channels (twice the number of parcels), explaining 65.4% of the variance. The PCA-transformed data were normalized to zero mean and unit variance before model training.

#### 2.3.3 HMM inference

HMM inference was conducted on one GPU NVIDIA A100 Tensor Core 40 Go. We conducted 5 inferences for*K*= 8 states. Model selection was based on both a quantitative criterion (i.e., minimize free energy) (Gottwald & Braun, 2020) and a qualitative criterion to identify a low-dimensional data representation that provides a good trade-off between fitness and complexity (Alonso & Vidaurre, 2023). That is, we compared inferences with different number of states by inspecting the spatio-spectral features of each state, that is the networks involved and the frequency content, and found that eight states resolved rich and specific spatio-spectral features across states in line with the toolbox recommendation and previous resting-state studies (Baker et al., 2014;Tibon et al., 2021).

### 2.4 Brain states analysis

#### 2.4.1 Spectral analysis

As shown inFigure 1, we multiplied the inferred parameters from the model with the unprepared source-space MEG data to retrieve the state time courses. Then, we conducted a multitaper analysis across the 1–45 Hz range to extract spectral features (Vidaurre et al., 2016,^2018^), resulting in subject- and state-specific spectra.

To enhance interpretability, we determined state-relevant frequency bands in a data-driven manner by running non-negative matrix factorization for each state separately. We asked for a decomposition into four frequency bands following the toolbox guidelines, and visually matched each dynamic state’s spectrum to its frequency band of interest (seeFigs. S3–S4, Supplementary Materials).

#### 2.4.2 Spatial analysis

We mapped brain states onto several resting-state networks. We obtained power maps in the spatial domain for each brain state and region by integrating the corresponding group-averaged spectrum within the corresponding frequency band of interest. The composition of each brain state was then obtained by multiplying the resulting power map with the volumetric overlap between each region and a 12-network atlas (Ji et al., 2019) (seeTables S1, Supplementary Materials). An illustration of these networks can be found inFigure S7, Supplementary Materials.

### 2.5 Neurocognitive analysis

To examine the relationship between resting-state MEG activity and lexical production (LP) performance across the lifespan, we employed Partial Least Squares (PLS) correlation analysis using the toolbox myPLS (https://github.com/MIPLabCH/myPLS) in MATLAB R2020b. PLS is a statistical technique identifying latent components, capturing coordinated changes between brain state features (*X matrix*) and cognitive (*Y matrix*) performances.

#### 2.5.1 PLS inference

As shown inFigure 1, the covariance matrix (X*Y*^T^*) undergoes singular value decomposition (SVD) to retrieve latent components. Each component is associated with a diagonal set of singular values (S), encoding the amount of shared information, and a set of brain (U) and cognitive (V) saliences, encoding the contribution of each feature. Statistical significance and robustness of saliences were assessed using 10,000 permutations and 1000 bootstrap resamples, respectively. Features with a high bootstrap sampling ratio (BSR ± 3), calculated as the salience weight over its bootstrapped standard deviation, indicate a robust contribution exceeding a 99% confidence interval (Krishnan et al., 2011).

#### 2.5.2 Data preparation

We examined the temporal and spectral brain state dynamics with three PLS models. As we expected similar latent components, we adjusted all models’ significance threshold at the False Discovery Rate (FDR).

*(i) Model 1: Temporal domain (within states).*For each subject and state, we calculated four metrics. The first two metrics characterize the duration of state activations: Fractional Occupancy (FO), the fraction of total time spent in a state; Lifetime (LT), the mean duration when a state is active. The last two metrics characterize the frequency of state activations: Interval Lifetime (INT), the mean duration between successive activations of the same state; Switching Rate (SR), the mean number of state activations per second.

*(ii) Model 2: Temporal domain (between states).*For each subject, we computed the probability that each state gets active after another, resulting in a state-by-state transition probability matrix. To ensure relevancy, we excluded self-self transitions in our calculations.

*(iii) Model 3: Spectral domain.*We concatenated the power spectra for each subject and state (dimension: subjects*by*states*by*parcels*by*frequencies). We limited our analysis to the top 20 parcels to ensure relevance, showing each state’s most extensive group-level activity.

For the three models, cognitive features were preprocessed in the following steps. First, we regressed out the following covariates: sex, total intracranial volume (TIV), MMSE score, and the number of time points contained in the preprocessed MEG recording of each subject. Second, we quantile-normalized each cognitive score to improve Gaussianity in line with previous work (Pur et al., 2022). Third, we added age-related information as follows:

Given the nonlinear age trajectory reported in MEG studies (Gula et al., 2021), we generated two piecewise polynomials that we included as separate variables in our models. The quadratic term used to generate the polynomials was centered at 55 years old based on converging evidence in different imaging modalities (Dohm-Hansen et al., 2024;Guichet, Roger, et al., 2024;Park & Festini, 2016;Roger et al., 2023) and matched to the original scale of the age variable. As shown inFigure 1, the first polynomial function represented the ascending part of an inverted U-shape trajectory and plateaued after age 55 years. Complementarily, the second polynomial function represented the descending part and accelerated after age 55 years. If our PLS model maximizes the covariance with a linear age trajectory, then we expect the two functions to have saliences of similar magnitudes but with the opposite sign. In contrast, a quadratic trajectory would yield saliences of similar magnitudes and signs. In sum, this approach allowed us to account, in a data-driven manner, for the optimal age-related trajectory within the subspace defined by linear and quadratic trajectories.

## 3 Results

### 3.1 Dynamic brain states

Leveraging the Hidden Markov Model approach, we modeled resting-state MEG activity and identified eight brain states whose activations wax and wane over time. Consistent with our hypothesis, we observed that DMN activity and suppression are crucial elements of time-varying functional activity. As shown inFigure 2, we observed that they are spectrally coupled within the 1–8 Hz and 1–25 Hz frequency range, respectively, in the anterior-ventral axis (states 1–7), where the DMN preferentially binds with the FPN network, and posterior-dorsal axis (states 2–8) where the DMN binds with lower-level circuitry.

**Fig. 2.**
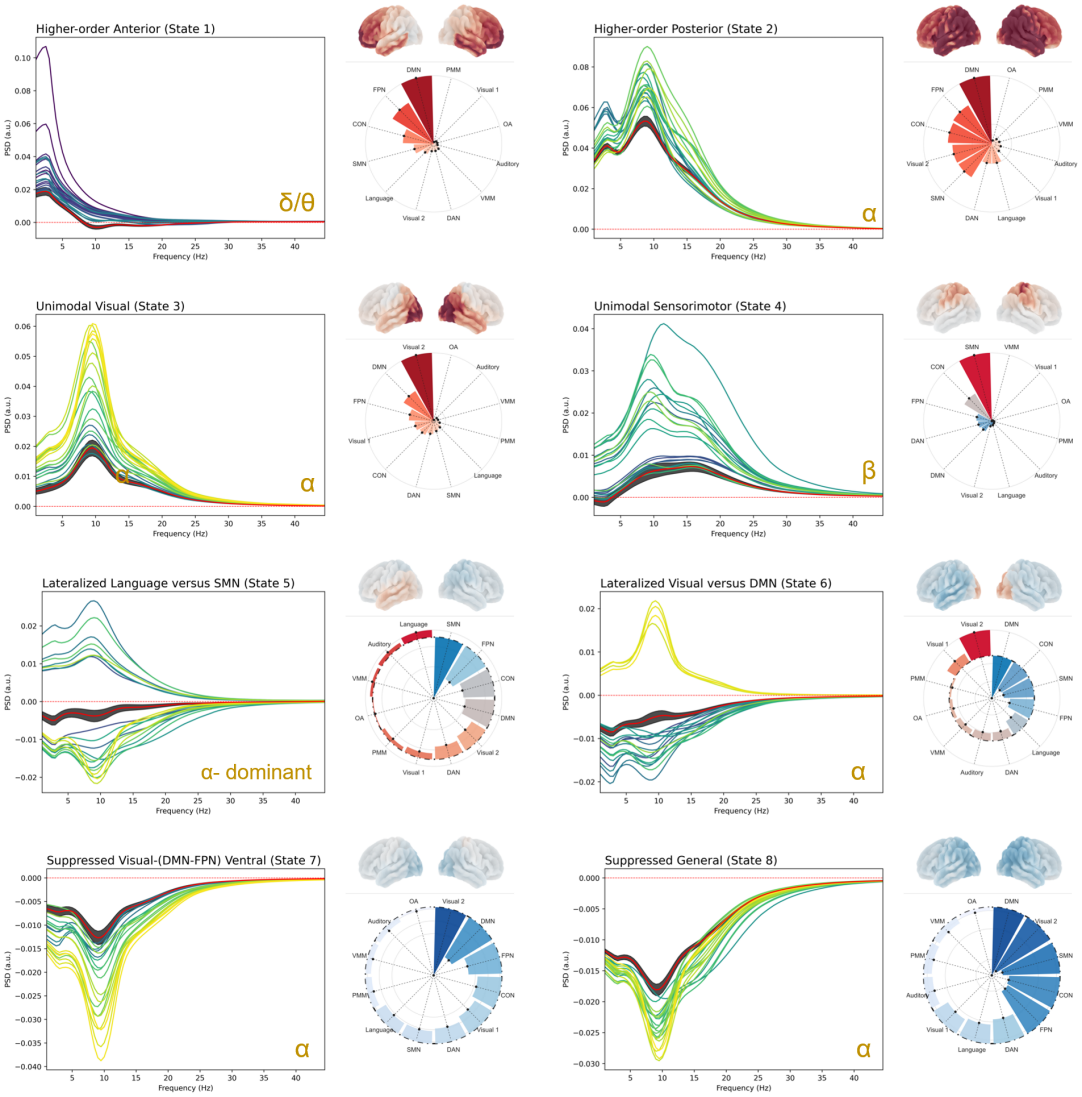
Dynamic spatio-spectral content of the eight brain states. Main plot illustrates the power spectra of the 20 channels with the largest departure from static activity, thus reflecting the dynamic features of each state. The colors of each channel follow their spatial location (yellow = posterior, green = middle, purple = anterior). The red line represents the mean power across all channels and participants. The black strip is the variance among channels.**Brain plot.**The group-level spectrum of each state is integrated into the spatial domain (red = activation; blue = suppression).**Radar plot.**Resting-state network topography (red = positive contribution; blue = negative contribution). For additional details, seeTable S8, Supplementary Materials. DMN (Default Mode), FPN (Fronto-Parietal), CON (Cingulo-opercular), DAN (Dorso-Attentional), SMN (Sensorimotor), VMM/PMM (Ventro-/Posterior Multimodal), OA (Orbito-Olfactive).

Below, we describe the group-level spatio-spectral features of each state, beginning with the states that show the largest oscillatory changes compared with the time average (seeFig. S1–S2, Supplementary Materials).

#### 3.1.1 Higher-order states

State 1 implies high activity in the delta/theta (δ/θ) band (~1–7 Hz). This state engages medial and lateral frontal areas of the DMN and FPN networks, respectively. In comparison, state 2 involves an increase in oscillatory activity across a wider 1–25 Hz frequency range, with prominent spectral peaks in the delta and alpha (α) bands. This state engages temporoparietal areas primarily in the lateral and posterior parts of the DMN, FPN, and CON networks.

#### 3.1.2 Unimodal states

State 3 is an alpha-band powered state with increased oscillatory activity in visuo-occipital areas. In comparison, state 4 shows increased beta-band (β) pre-/post-central sensorimotor activity.

#### 3.1.3 Lateralized states

States 5 and 6 are characterized by opposite oscillatory activity between the two hemispheres, showing mild positive or negative changes in the alpha band. State 5 engages left-hemispheric language-auditory circuitry at the expense of dense right-hemispheric sensorimotor-visual activity. In contrast, state 6 engages right-dominant visuo-occipital circuitry at the expense of left temporal DMN activity.

#### 3.1.4 Suppressed states

States 7 and 8 exhibit a mild suppression of alpha activity relative to the average over time. State 7 specifically engages more ventral posterior regions, while state 8 is more spatially distributed across a wider 1–25 Hz frequency range, mirroring the network and spectral characteristics of state 2.

### 3.2 Neurocognitive analyses

Using the Partial Least Squares (PLS) analysis, we sought to uncover how brain state features relate to changes in lexical production performance across the lifespan. We specifically examine temporal characteristics (duration and frequency of state activations), transitions between states, and spectral characteristics.

In line with our hypothesis, we consistently found (i) a**domain-general component**denoting a slight acceleration in cognitive control deficits beyond midlife primarily underpinned by changes in temporal dynamics (seeFig. 3A, B); (ii) a**semantic-specific component**denoting an inverted U-shaped trajectory across the lifespan, associated with enhanced semantic access and spectral changes (seeFig. 3C, D). Model diagnostics are reported inFigure S6, Supplementary Materials.

**Fig. 3.**
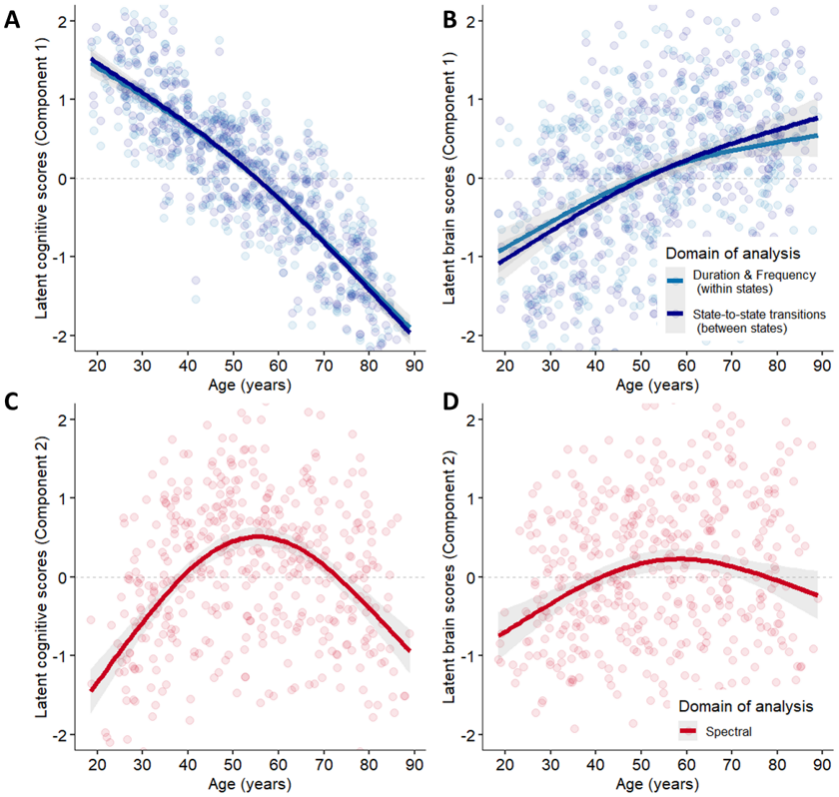
Latent age-related trajectories. Projection of the latent age-related scores associated with the cognitive and brain latent variables. (A, B) The first component correlates with the cognitive control aspects of lexical production and shows accelerated cognitive decline beyond age 60 years in the temporal models. (C, D) The second component correlates with the semantic/multitasking processes related to lexical production and peaks in midlife. Only the spectral model explains a significant amount of variance. The inflection point was fixed a priori to 55 years old (seeSection 2).

#### 3.2.1 Component 1: Temporal features reflect domain-general abilities

Figure 3Ashows that the first component negatively correlates with age, denoting more pronounced difficulties beyond age 60 years. This may primarily affect performances in tasks assessing fluid intelligence and picture-naming abilities and could be exacerbated by low education attainment (Table 2).

**Table 2.** Bootstrap sampling ratios (BSR) of education and cognitive variables for LC1.

Education and cognitive variables		Temporal model (within states)	Transition model (between states)	Spectral model
Education level (at age 30 years)		3.9	3.4	4.4
	*Average:*			
Naming *(generation/LTM//EF)*	*14.3*	11.9	13.2	17.7
Cattell *(general fluid abilities)*	*12.7*	11	12.1	14.9
Tip-of-the-tongue *(retrieval/EF)*	*8.8*	8.7	7.9	9.9
Verbal Fluency *(generation/LTM//EF)*	*7*	5.9	6.7	8.4
Story Recall *(LTM)*	*6.9*	6.1	6.6	7.8
Hotel task *(planning, multitasking)*	*5.7*	6.3	5.2	5.6
Sentence comprehension *(semantic/syntactic)*	*3.3*	3.4	*ns.*	3.5
Proverb *(semantic/EF)*	*ns.*	*ns.*	*ns.*	*ns.*

Only salient (±3) bootstrap sampling ratios (BSR) are presented. These values are positively correlated to the trajectory shown inFigure 3Afor the temporal/transition models, and 3C for the spectral model. ns (not significant).

Interestingly, temporal changes within and between brain states explained most of the total shared variance (86.93% and 84.3%;*p*_FDR_< .001) compared with the spectral model (58.75%;*p*_FDR_< .001). This suggests that temporal dynamics adequately capture the lifespan trajectory of cognitive control performances involved in lexical production. Moreover, we note that some temporal changes may work to mitigate control deficits as a reduction in these changes beyond midlife concurs with a slight acceleration in cognitive decline (seeFig. 3A). Thus, we primarily focus on the salient temporal features associated with this first component. Results on the spectral model are available inSupplementary Materials.

##### 3.2.1.1 Duration and frequency (within states)

**Fig. 4.**
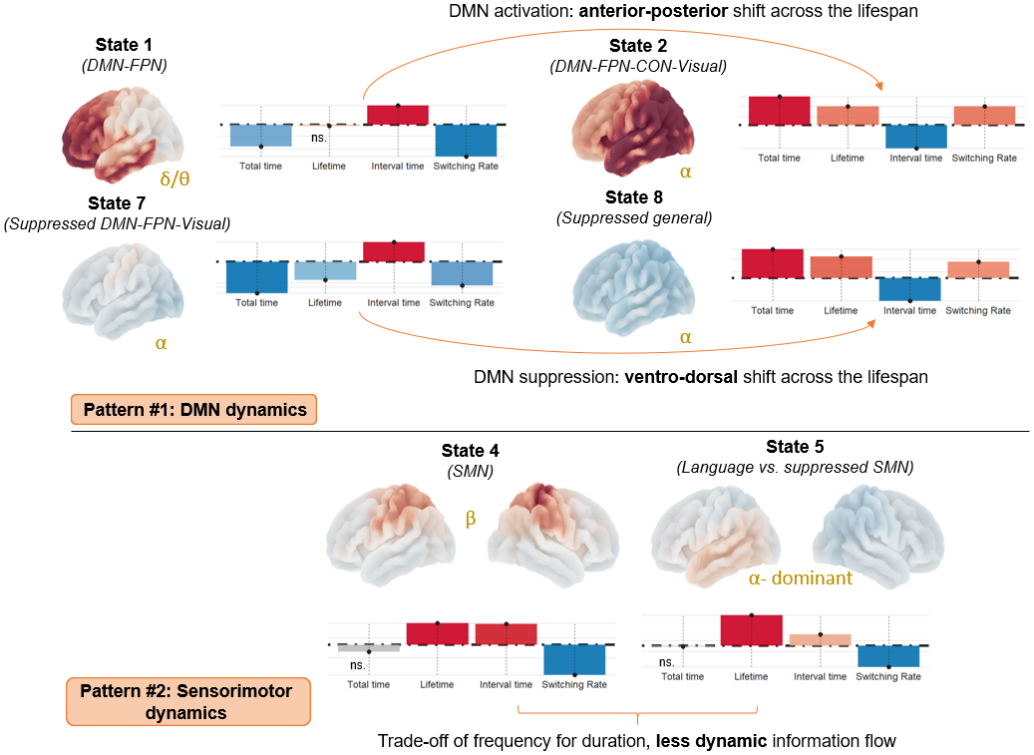
Bootstrap sampling ratios (BSR) for temporal metrics. We report two patterns contributing to the lifespan trajectory shown inFigure 3Band age-related deficits in domain-general naming processes as reported inTable 2/Figure 3A. The first pattern represents an anterior-posterior DMN activity and ventral-dorsal DMN suppression shift. The second pattern represents a reduction of dynamic activity in sensorimotor cortices.**Brain plot.**The group-level spectrum of each state is integrated into the spatial domain (red = activation; blue = suppression). DMN (Default Mode), FPN (Fronto-Parietal), CON (Cingulo-opercular), ns (not significant), SMN (Sensorimotor).

Figure 4shows two main patterns regarding the duration and frequency of state activations contributing to the trajectories shown inFigure 3B: the first pattern involves states engaging DMN regions, and the second pattern involves SMN regions.

More specifically, the first pattern is characterized by an increase in overall time spent activating the states (state 2; FO = 11.5; state 8; FO = 11.7) where the DMN binds with both the attentional (FPN, CON) and sensory-visual networks (SMN, Visual2). This increase reflects shorter interval times between activations (INT = -9.3/-9.3), more frequent state visits (SR = 7.6/6.5), and longer activations (LT = 7.6/8.8). For clarity, we now refer to states 2 and 8 as older-like DMN-FPN states. Interestingly, this covaries with decreased overall time spent activating the states (state 1; FO = -12.1; state 7; FO = -16.6) where the DMN binds with the FPN and visual network, respectively, in prefrontal and ventral posterior areas. This decrease reflects longer interval times (INT = 10.7/10.4) and less frequent state visits (SR = -17.7/-12.6), but no significant changes in mean lifetime when the state is active (see alsoTable S1, Supplementary Materials for a summary). For clarity, we now refer to states 1 and 7 as younger-like DMN-FPN states.

The second pattern explicitly involves the sensorimotor network. Indeed, we observed that state 4, when the sensorimotor network is active, and state 5, when auditory-language inputs accompany sensorimotor suppression, have more sustained activations (LT = 6.9/12.7) but less frequent visits (SR = -9.6/-8.8) and longer interval times between state visits (INT = 6.6/4.7). Given that the overall time spent in these states does not change significantly, this alteration may be interpreted as a trade-off between the frequency and duration of activation.

In summary, our findings suggest an anterior-to-posterior and ventral-to-dorsal shift in the brain states’ temporal dynamics, all engaging the DMN. DMN-FPN state activations on the anterior (state 1) and ventral (state 7) ends get more infrequent with age (although not shorter), whereas DMN-FPN state activations on the posterior (state 2) and dorsal (state 8) ends get both more frequent and longer. Moreover, it seems that sensorimotor activation may become more inflexible with advancing age as the duration of state activations increases, but their frequency decreases. A thorough interpretation of these patterns is proposed in the discussion section.

##### 3.2.1.1 State-to-state transitions (between states)

When considering successive state-to-state transitions, our results revealed a similar anterior-posterior and ventral-dorsal shift in how information flows is re-orchestrated, explicitly highlighting critical alterations in sensorimotor processing.

**Fig. 5.**
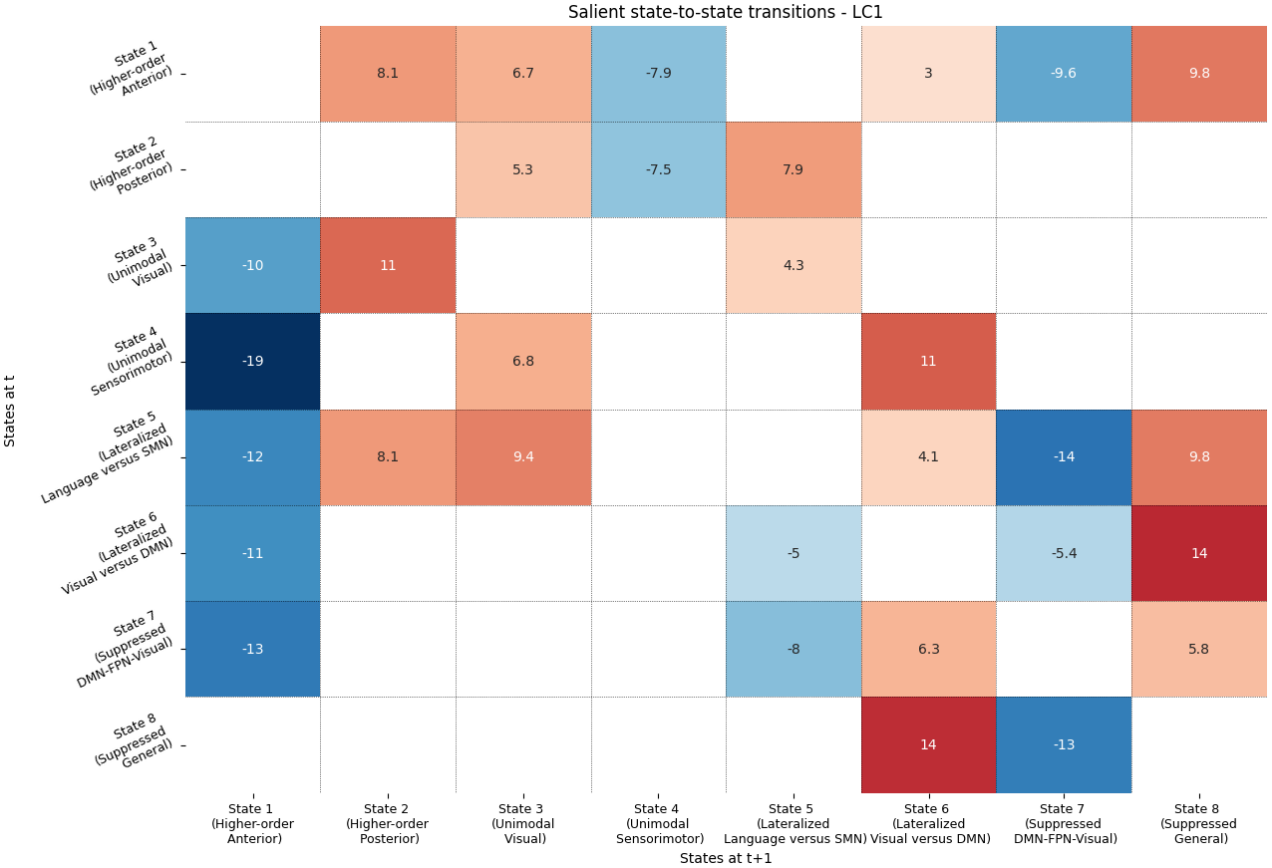
Bootstrap sampling ratios (BSR) for state-to-state transitions. Each cell corresponds to state-to-state transitions from time t to t + 1. For example, the transition from the unimodal sensorimotor state (on the y-axis) to the higher-order anterior state (on the x-axis) is significantly reduced as age increases (BSR = -19). In other words, this transition is negatively correlated to the trajectory highlighted inFigure 3B. DMN (Default Mode), FPN (Fronto-Parietal), CON (Cingulo-opercular), SMN (Sensorimotor).

On the anterior and ventral ends,Figure 5shows reduced bidirectional information flow with younger-like DMN-FPN states with advancing age: (i) between the anterior state and activity in the beta band (transition states 4 to 1 = -19; states 1 to 4 = -7.9), (ii) between the ventral state and simultaneous auditory-language processing/sensorimotor suppression in the alpha band (transition states 5 to 7 = -14, states 7 to 5 = -8). The bidirectional information flow between younger-like DMN-FPN brain states is also reduced (states 1 to 7 = -9.6; states 7 to 1 = -13) in favor of more dorso-posterior alpha-band processing in state 8 (states 1 to 8 = 9.8; states 8 to 7 = -13). This is paralleled by preferential flow of sensorimotor information toward alpha-band activity in visuo-occipital areas (states 4 to 3 = 6.8, states 4 to 6 = 11). This information is then more likely to be processed in dorso-posterior DMN-FPN states (states 3 to 2 = 11; states 6 to 8 = 14). We also note increased transitions to these states directly after simultaneous auditory-language processing/sensorimotor suppression: states 5 to 2 = 8.1; states 5 to 8 = 9.8.

To further examine the state dynamics, we computed the most likely four-state cycle in early and older adulthood that contribute to domain-general abilities reported inTable 2.

**Fig. 6.**
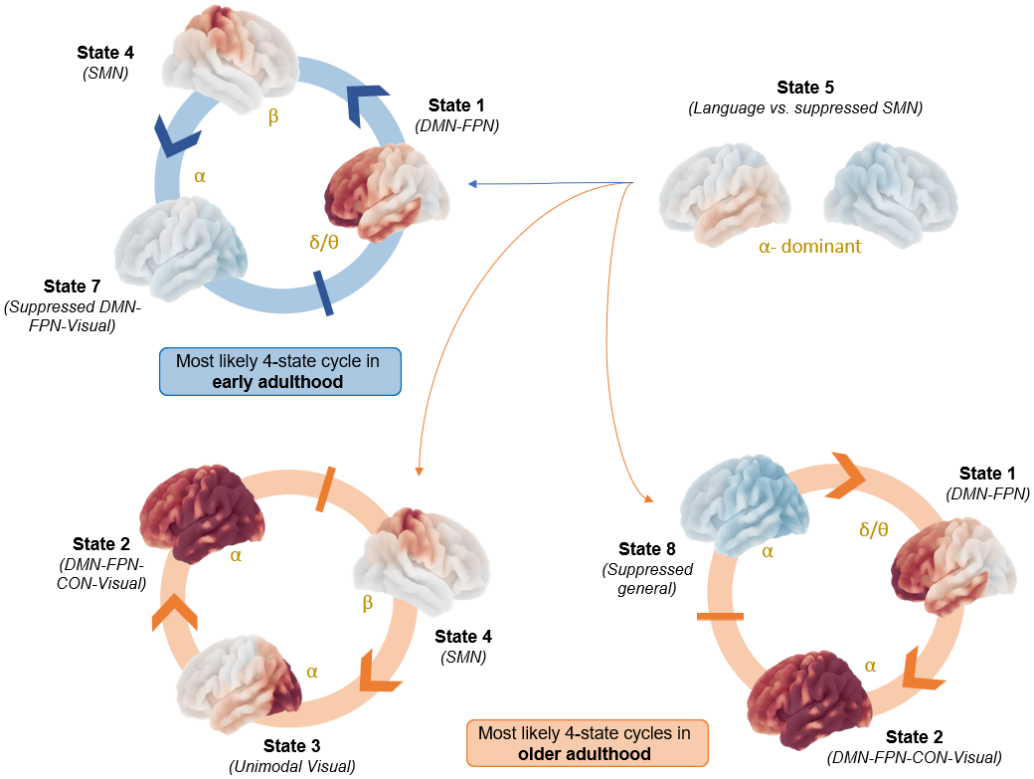
Most likely four-state cycles. These cycles correlate with the lifespan trajectory shown inFigure 3Band age-related deficits in domain-general naming processes as reported inTable 2andFigure 3A. The arrows from state 5 represent the time when this state joins in the cycle. For example, in early adulthood, state 5 is active before state 1, then follows state 4, and state 7. DMN (Default Mode), FPN (Fronto-Parietal), CON (Cingulo-opercular), SMN (Sensorimotor).

Figure 6shows that, in early adulthood, auditory-language inputs along are directly integrated in the anterior DMN-FPN state, before transmitting the information to sensorimotor cortices, and subsequent suppression in the ventral DMN-FPN state (BSR = 6.27). In older adulthood, we found two cycles that mostly operate in the alpha range: (i) auditory-language inputs are integrated in the DMN-FPN states along an anterior-posterior route (BSR = 5.68); (ii)) auditory-language inputs are processed in the sensorimotor cortices and integrated in the higher-order dorso-posterior state via alpha-band visuo-posterior activity (BSR = 5.67).

#### 3.2.2 Component 2: Spectral features reflect semantic-specific processes

The second latent component was significant across all models, but only spectral information explained a significant portion of the variance (28.68%;*p*_FDR_< .001).

**Fig. 7.**
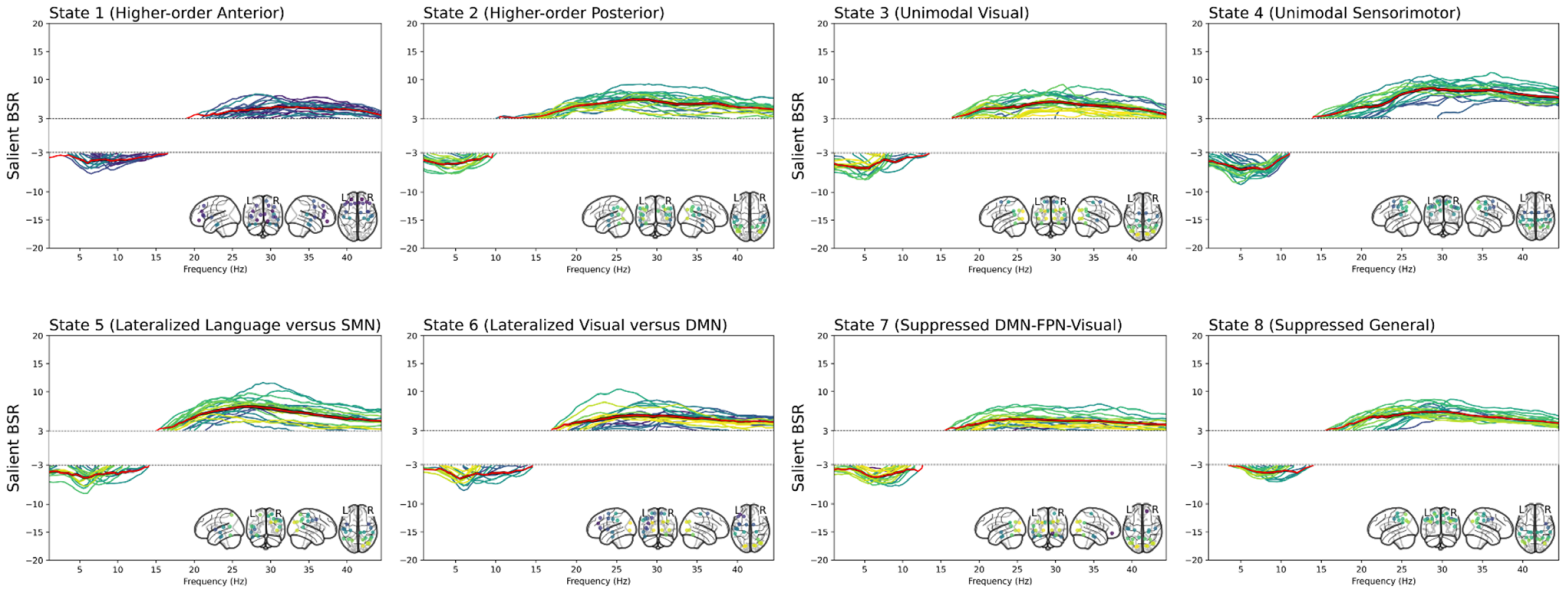
Bootstrap sampling ratios (BSR) in the spectral domain. These changes correlate with the lifespan trajectory shown inFigure 3Dand age-related increases in domain-general naming processes and education levels as reported inTable 2/Figure 3C. The colors of each channel follow their spatial location (yellow = posterior, green = middle, purple = anterior). Red is the mean salient BSR value; the gray strip is the standard error. The white band in the range y = -3 to y = 3 masks BSR values below the significance threshold. DMN (Default Mode), FPN (Fronto-Parietal), CON (Cingulo-opercular), SMN (Sensorimotor).

This component captured semantic aspects of lexical production such as semantic abstraction (BSR_proverb task_= 10) and comprehension (BSR = 4) but also highlighted the multitasking/self-monitoring processes, as measured by the Hotel Task (BSR_hotel task_= 9.2), which could be recruited for maintaining verbal fluency (BSR = 9.2) and naming abilities (BSR = 5.7). We also note better long-term memory performances (BSR = 4.3), which continue to suggest the exploitation of stored knowledge in semantic memory for lexical production.Figure 7shows that this cognitive outcome peaks in midlife and positively correlates with education attainment (BSR = 5.2). At the brain level, this mapped onto a general increase in the upper part of the spectrum (>13 Hz) across all states and a release of activity in the lower part (<13 Hz).

## 4 Discussion

As individuals age, lexical production, including word generation and retrieval, may increasingly demand cognitive control to manipulate and access the semantic store efficiently (Hoffman & Morcom, 2018). While semantic repositories significantly expand with age, cognitive control decline may lead to longer naming latencies, typically beginning around midlife. Thus, the present study aims to understand the neurocognitive mechanisms that delay the onset of lexical production decline with age, focusing on the role of semantic control in preserving language function.

Research has primarily utilized imaging techniques with low temporal resolution, such as fMRI, to investigate these mechanisms (Alves et al., 2023;Martin et al., 2022). Consequently, more is needed to understand the brain-wide changes in spontaneous oscillatory dynamics accompanying the onset of lexical production decline across the lifespan. To address this gap, our study leveraged recent machine learning advances for modeling the magnetoencephalographic (MEG) resting-state activity of 621 participants aged 18–88 years from the CamCAN cohort (Cam-CAN et al., 2014), and investigated relationships with cognitive performances in 427 of them.

Importantly, our study reaffirms that midlife is a turning point for spontaneous network oscillatory dynamics underlying language performance, consistent with previous MEG (Gula et al., 2021;Kupis et al., 2021), time-averaged fMRI (Guichet, Banjac, et al., 2024), and diffusion-weighted imaging (DWI) (Guichet, Roger, et al., 2024;Kljajevic & Erramuzpe, 2019). This further underscores the importance of studying the middle-aged brain from a multimodal perspective (Lachman, 2015;Park & Festini, 2016) as this period of life could be “prognostic of future cognitive outcomes” (Dohm-Hansen et al., 2024).

### 4.1 Spatial and spectral specificity of DMN-FPN dynamics decreases with age

From a neurofunctional perspective, we identified recurrent dynamic networks or*states*at the group level, providing an integrated spatial, temporal, and spectral description of the brain’s functional dynamics at rest (Fig. 2). The neurophysiological signature of these states is consistent with recent work demonstrating that neural dynamics at rest distinguish key cortical territories including “unimodal sensory regions (states 3 and 5), precentral motor areas (state 4), and spatially distributed transmodal systems (states 1 and 2)” (Royer et al., 2025).

While the spatial topographies of certain brain states are strongly similar to low-level fMRI resting-state networks, areas of the Default Mode Network (DMN) are distributed within four states, reflecting its synchronization with multiple networks across frequency bands. This supports that DMN oscillations represent a distributed pattern of increased power aiding in the coordination of information flow across the brain (de Pasquale et al., 2018;Van Es et al., 2023;Vidaurre et al., 2018).

Relatedly, our results align with previous studies reporting a dissociation along the anterior-posterior axis when DMN drives higher-order states’ activations (Vidaurre et al., 2018) and the ventro-dorsal axis when DMN activity decreases alongside visuo-occipital processing (Tibon et al., 2021). In early adulthood, DMN-FPN activity straddles the prefrontal-temporal cortex in the delta/theta frequency range (state 1) and couples with DMN-FPN-Visual suppression in ventral occipital areas in the alpha range (state 7). In older ages, we note more integrated temporoparietal DMN activity with attentional and visuo-sensorimotor subsystems in a broader alpha-dominant 1–25 Hz frequency range (state 2), which is paralleled by spatially diffuse suppression in dorso-posterior areas of the same networks.

This echoes accounts of dedifferentiation usually reported in fMRI studies whereby older adults are less selective in the recruitment of regions supporting cognitive functioning (Deery et al., 2023). Similarly, our study suggests that older adults are less “spectrally selective.” That is, while younger adults operate on distinct rhythms for DMN-FPN activation and suppression, respectively, older adults operate a broader rhythm for both, thus reflecting more spatially diffuse dynamics in an alpha-dominant band only. This loss of spectral specificity is in line with our hypothesis that older adults enhance alpha-band processing for increasing top-down control during semantic search processes (Zioga et al., 2024). As discussed below, this is generally associated with age-related cognitive decline but could also reveal an alpha-dominant compensatory mechanism

### 4.2 Enhanced dorso-posterior DMN-FPN temporal dynamics: Alpha-based compensation?

Indeed, changes in the temporal dynamics explained as much as 85% of the age-related trajectory associated with lexical production performance (Section 3.2), particularly considering the decline in fluid abilities, word generation, and retrieval. More specifically, anterior and ventral information flow in the younger-like DMN-FPN states in the delta/theta and alpha range becomes less frequent with age, with fewer transitions to these states. In contrast, dorso-posterior alpha-dominant information flow in the older-like DMN-FPN states shows the opposite pattern. On a general note, this further suggests that DMN-FPN temporal dynamics are key to the age-related mechanisms underlying lexical production decline. This also demonstrates that temporal features best predict domain-general aspects of production, which aligns with the idea that inhibitory control is time dependent as mentioned in the introduction (Courtney & Hinault, 2021).

While the shift toward dorso-posterior DMN-FPN dynamics strongly correlates with age-related cognitive decline, greater recruitment of these alpha-based states could partially represent a compensatory mechanism that upholds, albeit inefficiently, DMN-FPN flexibility.

Indeed, we found that cognitive decline accelerates when temporal changes plateau beyond midlife (refer to the latent trajectories inFig. 3A, B). Thus, compensatory and maladaptive mechanisms may coexist in the context of cognitive decline (McDonough et al., 2022). In that regard, our correlational results alone cannot fully discern the temporal changes contributing to such compensation from those reflecting cognitive decline. For example, this shift could compromise cognitive flexibility (Battaglia et al., 2020), or enhance the cross-talk between the DMN and lower-level circuitry in these states under a similar brain rhythm to preserve lexical production. In line with the latter, recent work suggests that increased dwell time in older adults could signal an exploitative search (Spreng & Turner, 2021) and promote selective access to knowledge systems (Klimesch, 2012). This is further discussed in the exploration–exploitation section below.

### 4.3 Potential evidence of an exploration–exploitation shift

Previous studies may provide further evidence supporting a broader exploration-to-exploitation shift during healthy cognitive aging (Brown et al., 2022;Spreng & Turner, 2021). According to this framework, older adults perform best when exploiting accumulated semantic knowledge, for example, in inferential naming tasks that rely on semantic/episodic associations as opposed to traditional picture naming paradigms (Fargier & Laganaro, 2023). This is especially relevant to our results, considering that increased recruitment of posterior regions has been reported in older adults for object naming (Hoyau et al., 2017) and semantic processing (Lacombe et al., 2015).

Moreover, the anterior-posterior shift in higher-order cognitive states (from states 1 to 2;Fig. 4) echoes a similar spatio-functional dissociation in the form of control processes relevant for goal-directed behavior (Mansouri et al., 2017). Lateral and medial parts of the frontopolar cortex, as found in state 1, may facilitate goal selection and monitoring which authors associate with an exploration drive. In contrast, more posterior cortical regions may be geared toward implementing cognitive control to optimize the current task, which authors associate with an exploitation drive. Thus, the anterior-to-posterior shift in DMN temporal activity seems to have a strong cognitive justification, and we propose to interpret this shift as evidence of a transition from an exploration-driven to an exploitation-based strategy to support lexical production performance.

Although metabolic underpinnings have not been addressed in this study, our results also corroborates the predictions made by the SENECA model (Guichet, Banjac, et al., 2024) and are in line with recent work showing that older adults have an overall smaller metabolic energy budget with the largest proportion allocated to posterior regions (Deery et al., 2024). Indeed, the additional engagement of the Cingulo-Opercular Network (CON) network in more posterior states (Section 3.2) supports the hypothesis that older adults gradually adopt a more “energy-efficient,” bottom-up form of cognitive control through increased transitions between the DMN (vmPFC & PCC) and the CON (fronto-insular and anterior cingulate cortices) (Brown et al., 2022;Dosenbach et al., 2024;Jilka et al., 2014).

### 4.4 Sensorimotor information is redirected along a posterior route with age

Relatedly, in older adulthood, the shift toward more dorso-posterior processing was associated with crucial alterations in sensorimotor dynamics, consistent with claims that the sensorimotor network shapes the dynamic resting-state activity across the lifespan (Kong et al., 2021;Sastry et al., 2023).

Interestingly, our state-to-state transition results support distinct spatio-spectral paths for integrating auditory-language inputs across the lifespan, and these paths are correlated with domain-general changes in naming abilities. In early adulthood, these inputs are integrated in the anterior DMN-FPN and are afferent to the sensorimotor cortices, suggesting spectrally specific higher-order integration in the low delta/theta range to prepare articulatory-motor beta-range activity. In comparison, declines/inefficient compensation in domain-general cognitive functioning in older adults was associated with two paths.

(i) The first path involves a similar integration in anterior DMN-FPN state but contingent to greater cortex-wide alpha-dominant suppression of activity, followed by additional and more alpha-dominant dorso-posterior integration where the DMN interacts with lower-level circuitry. This could highlight the need for additional, yet inefficient, top-down processing more posteriorly as discussed previously, and could also reflect greater DMN-FPN coupling as predicted by the DECHA model (Spreng & Turner, 2019).
(ii) The second path is more indirect: mobilizing sensorimotor processing to redirect the information flow toward alpha-band visuo-posterior activity, which then mediates integration in the higher-order dorso-posterior state. Such acute engagement of the visuo-occipital activity could facilitate the integration of auditory-language inputs in older adulthood (Gonzalez Alam et al., 2024), with this integration being regulated by sensorimotor activity. The key role of the SMN is consistent with recent teamwork suggesting that more energy is being allocated to auditory-SMN connectivity beyond midlife (Guichet et al., in prep).

### 4.5 Sensorimotor information is less dynamic with age

Sensorimotor information, whether active (state 4) or suppressed when auditory-language processing occurs (state 5), was less dynamic with age as shown by longer interval between successive state activations and longer dwell time when the state is active (refer toFig. 4).

Indeed, with advancing age, the SMN stops integrating information coming from both higher-order states, specifically reducing bidirectional flow with younger-like DMN-FPN activity anteriorly. This further reinforces that the SMN plays a crucial role in regulating the transition from higher-order anterior processing to dorso-posterior processing with age, serving as either an afferent or efferent pathway, respectively. This also highlights that the cognitive control circuitry associated with word generation and retrieval crucially depends on interactions with pre- and post-central areas of the sensorimotor network in younger adults which optimize exploratory-driven goal selection (Fine & Hayden, 2022).

### 4.6 Spontaneous low gamma-band activity gateways semantic memory-guided control inputs

More marginally, our model highlighted a semantic-specific component by faster oscillatory rhythmic activity (13–45 Hz) in midlife and associated with enhanced semantic access and the multitasking aspect of control processes. This could highlight the enhanced semantic access, which relies on the bottom-up aspect of executive functioning suited to apply existing knowledge to real-life scenarios (i.e., exploitation-based mentation). Indeed, the Hotel task employed in this study appears to be a more ecologically valid assessment (Milia-Argeiti, 2022) than a standardized, distraction-free environment typically used to perform the Cattell task (i.e., to assess fluid reasoning). At the brain level, this is consistent with gamma power (30–150 Hz) being the main physiological basis of bottom-up information processing and could reflect the activation of local functional networks supporting semantic representations (Courtney & Hinault, 2021;Jensen et al., 2014). However, this result warrants further investigation as low gamma-band activity in our study is limited to 45 Hz and may be subject to interpretation limitations inherent to time-delay embedding (Rossi et al., 2023;Vidaurre et al., 2018).

### 4.7 Limitations of the study

Other methodological limitations include the use of cross-sectional data, which precludes direct inferences about the aging process, and the lack of cognitive reserve proxies beyond education levels. The Hidden Markov Model approach also holds certain assumptions, such as the a priori specification of the number of states and a Gaussian observation model, which may oversimplify the underlying network dynamics. Regarding the Partial Least Squares models, improving the fit between spectral information and cognitive outcomes may require examining cross-frequency couplings, given their crucial role in integrating, coordinating, and regulating neuronal activity (Palva & Palva, 2018). Additionally, we mention that we defined the turning point at age 55 years, fundamentally limiting the range of potential age trajectories explored during model inference. However, we argue that this approach arguably offers a less biased methodology for modeling age-related effects than discrete age groups and was based on previous teamwork which identifies midlife as a crucial turning point for lexical production performances (Guichet, Roger, et al., 2024). Accordingly, future work could fix the quadratic term at different ages following the study hypotheses or explore alternative unsupervised techniques better suited to explore nonlinear relationships with age. More complex models that relax Gaussianity and linearity assumptions, such as deep learning approaches, could provide additional insights. Future work should also explore how age-related changes reported in this study compare with task-related oscillatory dynamics.

## 5 Conclusion

This study aimed to elucidate the time-varying neurofunctional mechanisms involved in preserving lexical production across the lifespan. Our findings highlight two coupling patterns between DMN-FPN activation and suppression: a spectrally diverse coupling along the anterior-ventral axis in younger adults and an alpha-dominant coupling along the posterior-dorsal axis in older adults. Specifically, when information flow with mediolateral prefrontal areas is compromised, older adults may preferentially engage posterior regions to enhance the cross-talk between higher- and lower-level circuitry. Our results further highlight the crucial role of sensorimotor processing in this age-related reorganization, potentially facilitating auditory-motor integration in higher-order posterior cortices through alpha-powered visuo-occipital activity. Overall, the onset of lexical production decline at midlife could reflect challenges in mobilizing alpha-dominant posterior circuitry, indicating a greater reliance on a bottom-up, exploitation-based form of cognitive control. We recommend further examination of the interplay between abstraction, control, and action systems to fully capture the mechanisms involved in preserving lexical production.

## Supplementary Material

Supplementary Material

## Data Availability

Post hoc data can be downloaded at:https://doi.org/10.5281/zenodo.12799096. Code is made publicly available at:https://github.com/LPNC-LANG/MEG_CAMCAN_2024

## Supplementary Materials

This includes the description of the eight neuropsychological tasks and supplementary results. Supplementary material for this article is available here:https://doi.org/10.1162/imag_a_00551.
