# Supplementary material for: Lifespan oscillatory dynamics in lexical production: A population-based MEG resting-state analysis
Source: Imaging Neurosci (Camb). 2025 Apr 28;3:imag_a_00551. doi: 10.1162/imag_a_00551 (PMC12319834; doi:10.1162/imag_a_00551)
Supplement: Supplementary Material [file imag_a_00551-supp.pdf]

Supplementary materials

Description of the 8 neuropsychological tests used to assess lexical production. Please refer to original CamCAN articles for more information on behavioral datasets [1,2].

Cattell: *Cattell Culture Fair Test: Complete nonverbal puzzles involving series completion, classification, matrices, and conditions* [3].

Hotel Task: *Perform simulated tasks of a hotel manager: write customer bills, sort money, proofread adverts, sort playing cards, alphabetize a list of names. Total time must be allocated equally between tasks; there is not enough time to complete any task* [4]. ***In this study, we applied a log transformation and subtracted it from 1 to stay consistent with a decrease as age increases (i.e., 1-log(x)).***

Picture Naming: *Name the pictured object presented alone (baseline), then when preceded by a prime object that is phonologically related (one or two initial phonemes), semantically related (low or high relatedness), or unrelated* [5].

Proverb: *Read and interpret three English proverbs* [6].

Sentence Comprehension: *Listen to and judge the grammatical acceptability of partial auditory sentences that begin with an ambiguous sentence stem (e.g., "Tom noticed that landing planes…") followed by a disambiguating continuation word (e.g., "are") in a different voice. Ambiguity is either semantic or syntactic, with empirically determined dominant and subordinate interpretations* [7].

Story Recall: *Listen to a short story, recall freely immediately after, then again after a delay, and finally answer recognition memory questions* [8]. *Delayed recall measure used here.*

Tip-of-the-Tongue (ToT): *Participants are asked to name famous faces and indicate if they know/don't know/or have a ToT* [9]. ***In this study, we subtracted the score from 1 to stay consistent with a decrease as age increases (i.e., 1-x).***

Verbal Fluency: *Mean of letter (phonemic) fluency and animal (semantic) fluency task. For the phonemic fluency task, participants have 1 minute to generate as many words as possible beginning with the letter 'p'. For the semantic fluency task, participants have 1 minute to generate as many words as possible in the category "animals"* [10].

References

1. Taylor, J. R. *et al.* The Cambridge Centre for Ageing and Neuroscience (Cam-CAN) data repository: Structural and functional MRI, MEG, and cognitive data from a cross-sectional adult lifespan sample. *NeuroImage* **144**, 262–269 (2017).
2. Cam-CAN *et al.* The Cambridge Centre for Ageing and Neuroscience (Cam-CAN) study protocol: a cross-sectional, lifespan, multidisciplinary examination of healthy cognitive ageing. *BMC Neurol* **14**, 204 (2014).
3. Cattell, R. B. & Cattell, A. K. Measuring intelligence with the culture fair tests. *Institute for Personality and Ability Testing* (1960).
4. Shallice, T. & Burgess, P. W. DEFICITS IN STRATEGY APPLICATION FOLLOWING FRONTAL LOBE DAMAGE IN MAN. *Brain* **114**, 727–741 (1991).
5. Clarke, A., Taylor, K. I., Devereux, B., Randall, B. & Tyler, L. K. From Perception to Conception: How Meaningful Objects Are Processed over Time. *Cerebral Cortex* **23**, 187–197 (2013).
6. *Dementia and Normal Aging*. (Cambridge Univ. Press, Cambridge, 1994).
7. Rodd, J. M., Longe, O. A., Randall, B. & Tyler, L. K. The functional organisation of the fronto-temporal language system: Evidence from syntactic and semantic ambiguity. *Neuropsychologia* **48**, 1324–1335 (2010).
8. Tulsky, D. S., Chiaravalloti, N. D., Palmer, B. W. & Chelune, G. J. The Wechsler Memory Scale, Third Edition. in *Clinical Interpretation of the WAIS-III and WMS-III* 93–139 (Elsevier, 2003). doi:10.1016/B978-012703570-3/50007-9.
9. Brown, R. & McNeill, D. The "tip of the tongue" phenomenon. *Journal of Verbal Learning and Verbal Behavior* **5**, 325–337 (1966).
10. Lezak, M. D., Howieson, D. B., Bigler, E. D. & Tranel, D. *Neuropsychological Assessment*. (Oxford University Press, Oxford New York Auckland Cape Town, 2012).

Supplementary results

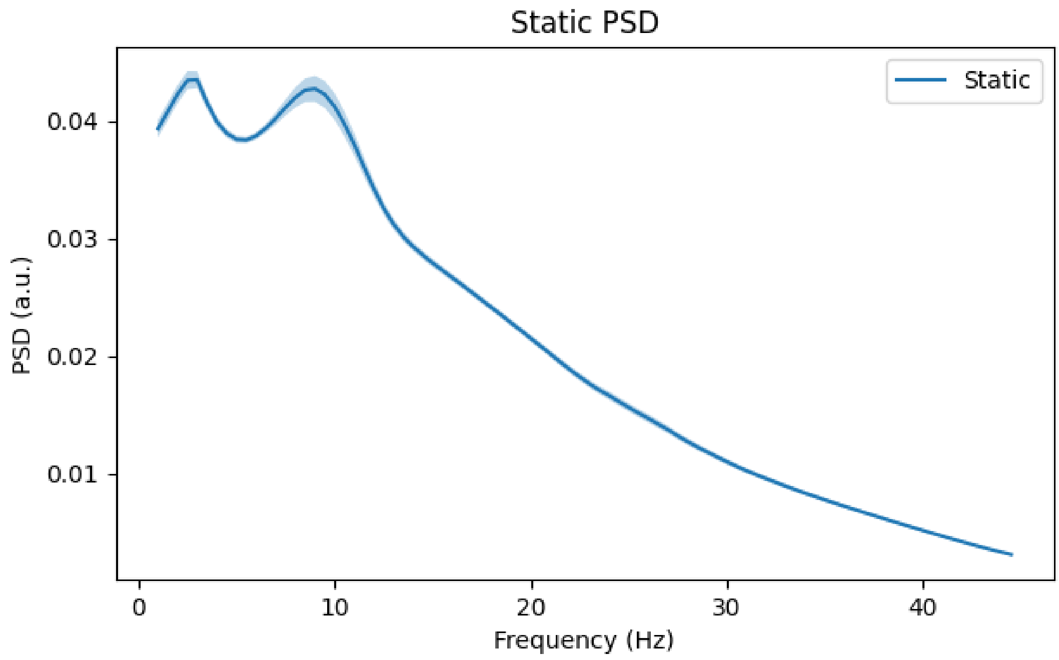

Figure S1. Group-level parcel-averaged static power spectrum. This represents the time-averaged spectrum weighted by the brain states' fractional occupancies. The more time spent in a state, the greater its influence on the static spectrum. *PSD=Power Spectral Density*

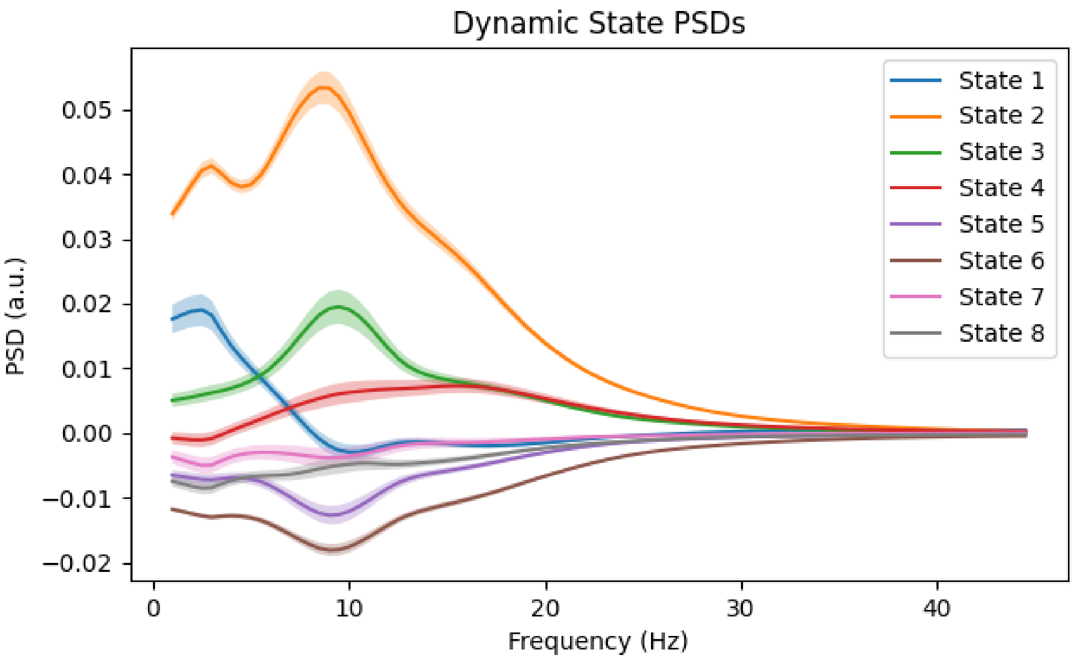

Figure S2. Group-level parcel-averaged dynamic power spectra. These spectra were obtained by subtracting the spectrum shown in Figure 1 from each state's static spectrum, thus reflecting state-specific dynamic activity. *PSD=Power Spectral Density*

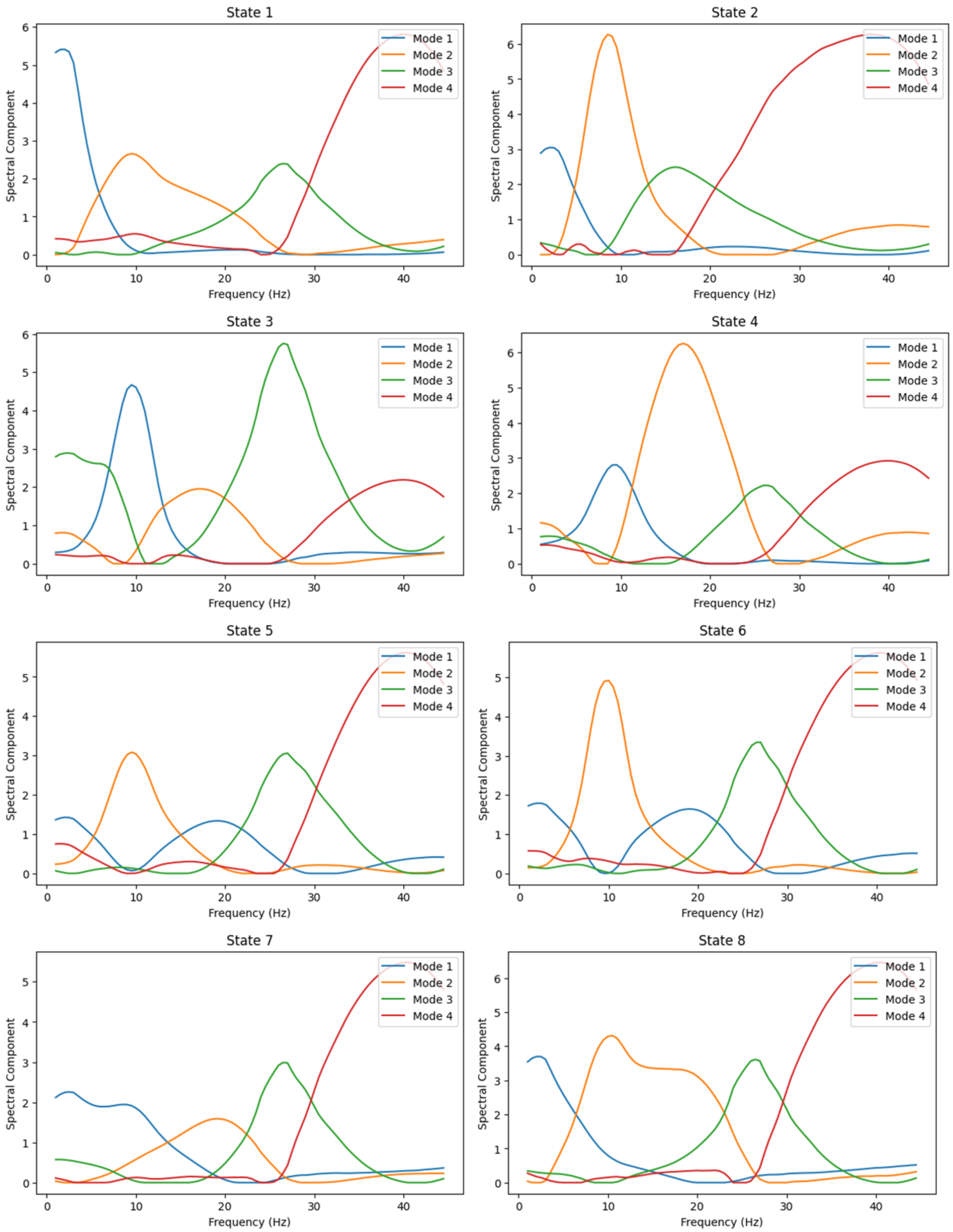

Figure S3. Spectral decomposition of each state. This decomposition was obtained by performing Non-Negative Matrix Factorization on the coherence spectra of each state.

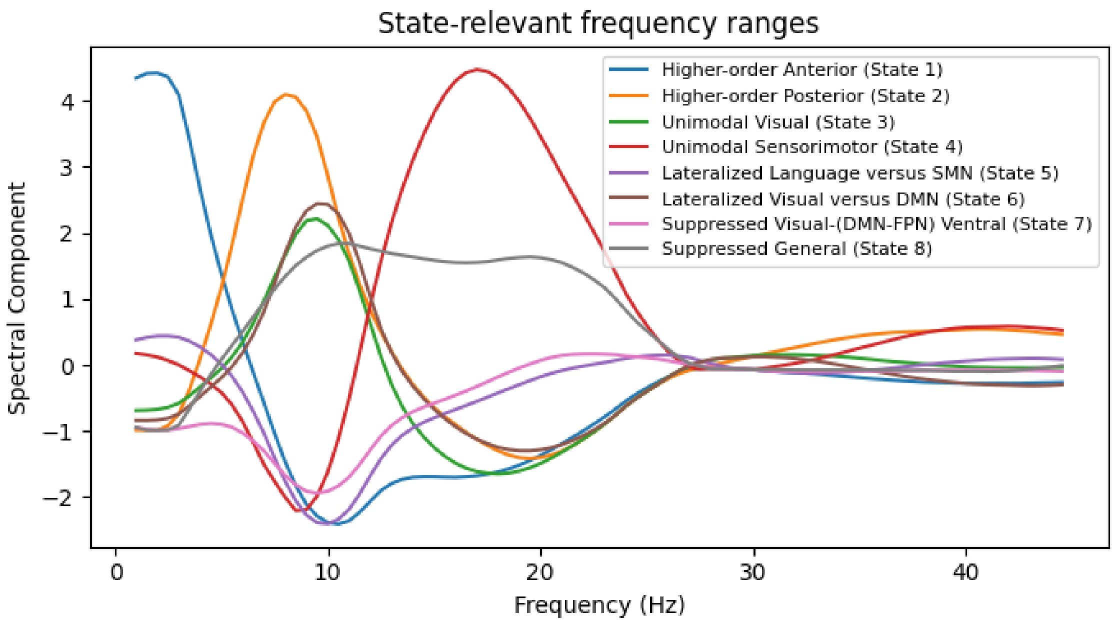

Figure S4. Group-level state-relevant frequency ranges. Following the decomposition shown in Figure S3, we matched one of the 4 component visually to the state's dynamic spectrum shown in Figure S1.

Brain state *(number – label)*	**Duration of state activations**		**Frequency of state activations**	
	Fractional Occupancy (FO)	***Mean Lifetime (LT)***	***Mean Interval time (INT)***	***Switching rate (SR)***
1- Higher-order Anterior	-12.1	*ns.*	10.7	-17.7
2- Higher-order Posterior	11.5	7.6	-9.3	7.6
3- Unimodal Visual	*ns.*	*ns.*	*ns.*	*ns.*
4- Unimodal SMN	*ns.*	6.9	6.6	-9.6
5- Lateralized Language vs. SMN	*ns.*	12.7	4.7	-8.8
6- Lateralized Visual vs. DMN	9	9.8	*ns.*	*ns.*
7- Suppressed DMN-FPN-Visual	-16.6	-9.6	10.4	-12.6
8- Suppressed General	11.7	8.8	-9.3	6.5

Table S1. Summary of the first latent component's bootstrap sampling ratios (BSR) in the temporal domain. Only salient (±3) BSRs are reported. *ns. not significant.* This table is illustrated in Figure 4 in the main text.

Results on the spectral PLS model

The first latent component explained 58.75% of the total shared variance ($p_{FDR} < .001$). Spectral changes were linear across the lifespan (*edf* = 1, *F* = 179.2) as opposed to the accelerating cognitive trajectory observed in our study. This suggests that spectral dynamics do not directly reflect cognitive control aspects of lexical production as far as our study is concerned.

However, such low performance could also be explained by methodological concerns: (i) setting an a priori inflection point at age 55 precludes the PLS model from exploring alternative trajectories that may better capture lifespan spectral changes, for example, with an inflection earlier or later in life. (ii) Cross-frequency couplings could better reflect the age-related cognitive trajectory. As shown in Figure S5, we observed oscillatory suppression in the lower and uppermost band (<8 Hz & >30 Hz), which could reflect theta-gamma phase-locking patterns previously associated with attention allocation [1], semantic [2], sensory and memory processes [3] during healthy aging [4]. More broadly, we found a release in alpha and beta-band power in visuo-occipital areas, which could indicate a trend towards a global inhibition release with aging (see the corresponding channels in yellow in Figure S5 below), together with increased activity in the ~8-25 Hz frequency range over the motor and premotor regions (see the corresponding channels in violet and green) which could indicate enhanced sensorimotor-related information flow across the cortex.

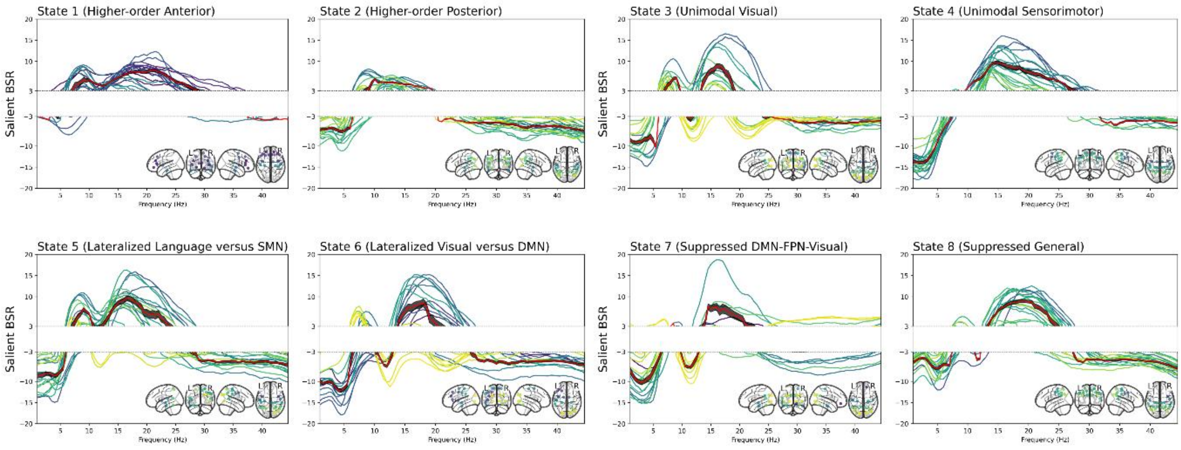

Figure S5. Bootstrap sampling ratios (BSR) in the spectral domain. The colors of each channel follow their spatial location (yellow = posterior, green = middle, purple = anterior). Red is the mean salient BSR value; the Grey strip is the standard error. The white band in the range y = -3 to y = 3 masks BSR values below the significance threshold.

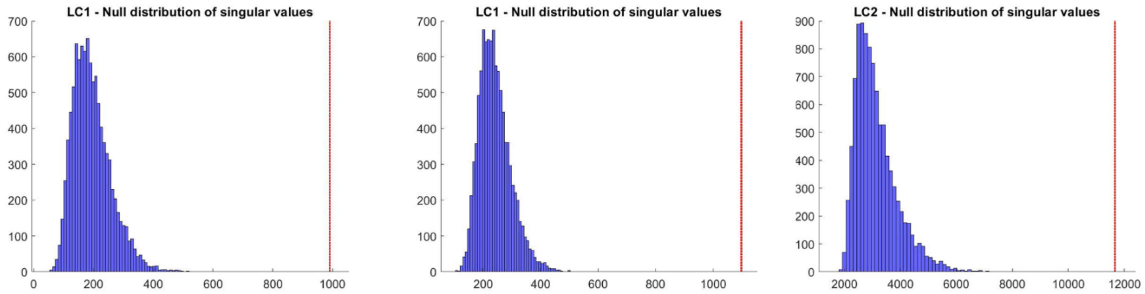

Figure S6. Partial Least Squares (PLS) model diagnostics. The null distribution of singular values following 10,000 permutations of the cognitive matrix for the first latent component of the temporal (left) and state-to-state transition (middle) models and the second latent component of the spectral model (left).

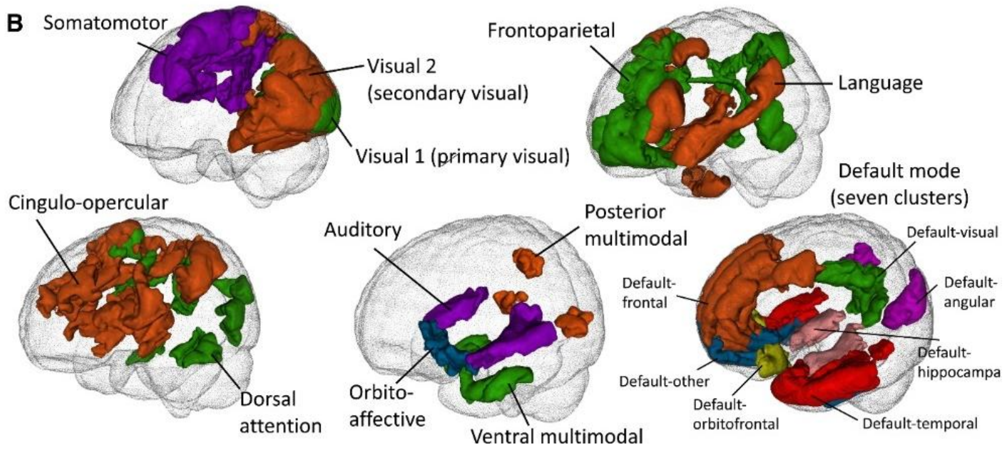

Figure S7. Reproduced illustration of the 12-network atlas used in the study. This was reproduced from Figure 1B in [5] and initially described in [6].

Resting-state networks	***State 1***	***State 2***	***State 3***	***State 4***	***State 5***	***State 6***	***State 7***	***State 8***
Visual 1	337,6	1216,79	1327,08	11,39	-100,21	775,65	-340,48	-368,81
Visual 2	710,12	4794,1	4728,8	143,75	-305,61	2346,65	-1153,33	-1419,98
SMN	1624,7	3581,19	-671,32	1910,64	-605,15	-993,18	32,94	-1780,98
CON	3067,31	4128,38	-334,64	789,18	-325,85	-1019,49	-391,4	-1715,08
DAN	689,54	2124,9	568,71	375,62	-140,86	-67,35	-219,43	-687,52
FPN	4943,76	4291,75	272,25	253,49	-387,7	-713,48	-689,06	-1732,52
DMN	7227,94	6668,94	463,85	27,5	-304,59	-1247,12	-1052,38	-2428,4
PMM	196,01	767,06	367,45	56,96	-44,74	61,24	-99,89	-213,32
Language	1253,79	2012,17	154,62	60,79	49,39	-431,11	-227,47	-620,94
Auditory	425,7	1019,85	159,04	63,45	32,45	-157,66	-83,8	-256,31
VMM	767,09	821,56	157,69	-55,03	19,89	-127,61	-133,9	-240,99
OA	399,96	329,8	-36,59	8,23	-6,55	-101,12	-42,85	-125,77

Figure S8. RSN-by-State map used for interpretation.

References

1. Courtney, S. M. & Hinault, T. When the time is right: Temporal dynamics of brain activity in healthy aging and dementia. *Progress in Neurobiology* **203**, 102076 (2021).
2. Mellem, M. S., Friedman, R. B. & Medvedev, A. V. Gamma- and theta-band synchronization during semantic priming reflect local and long-range lexical–semantic networks. *Brain and Language* **127**, 440–451 (2013).
3. Lisman, J. E. & Jensen, O. The Theta-Gamma Neural Code. *Neuron* **77**, 1002–1016 (2013).
4. Ehrhardt, N. M., Flöel, A., Li, S.-C., Lucchese, G. & Antonenko, D. Brain oscillatory processes related to sequence memory in healthy older adults. *Neurobiology of Aging* **139**, 64–72 (2024).
5. Zhao, B. *et al.* An atlas of trait associations with resting-state and task-evoked human brain functional organizations in the UK Biobank. *Imaging Neuroscience* **1**, 1–23 (2023).
6. Ji, J. L. *et al.* Mapping the human brain's cortical-subcortical functional network organization. *NeuroImage* **185**, 35–57 (2019).
